# The Environmental Pollutant Bromophenols Interfere With Sulfotransferase That Mediates Endocrine Hormones

**DOI:** 10.3389/fendo.2021.814373

**Published:** 2022-01-07

**Authors:** Zhihong Dai, Furong Zhao, Ying Li, Jing Xu, Zhiyu Liu

**Affiliations:** ^1^ Department of Urology, Second Hospital of Dalian Medical University, Dalian, China; ^2^ Research Department, Dalian Innovation Center of Laboratory Medicine Mass Spectrometry Technology, Dalian, China; ^3^ Research Department, Clinical Mass Spectrometry Profession Technology Innovation Center of Liaoning Province, Jinzhou, China

**Keywords:** bromophenols (BPs), endocrine hormones, sulfotransferase (SULT), inhibition, *in vitro-in vivo* extrapolation (IVIVE)

## Abstract

Bromophenols (BPs), known as an important environmental contaminant, can cause endocrine disruption and other chronic toxicity. The study aimed to investigate the potential inhibitory capability of BPs on four human sulfotransferase isoforms (SULT1A1, SULT1A3, SULT1B1 and SULT1E1) and interpret how to interfere with endocrine hormone metabolism. P-nitrophenol(PNP) was utilized as a nonselective probe substrate, and recombinant SULT isoforms were utilized as the enzyme resources. PNP and its metabolite PNP-sulfate were analyzed using a UPLC-UV detecting system. SULT1A1 and SULT1B1 were demonstrated to be the most vulnerable SULT isoforms towards BPs’ inhibition. To determine the inhibition kinetics, 2,4,6-TBP and SULT1A3 were selected as the representative BPs and SULT isoform respectively. The competitive inhibition of 2,4,6-TBP on SULT1A3. The fitting equation was y=90.065x+1466.7, and the inhibition kinetic parameter (K_i_) was 16.28 µM. *In vitro-in vivo* extrapolation (IVIVE) showed that the threshold concentration of 2,4,6-TBP to induce inhibition of SULT1A3 was 1.628 µM. *In silico* docking, the method utilized indicated that more hydrogen bonds formation contributed to the stronger inhibition of 3,5-DBP than 3-BP. In conclusion, our study gave the full description of the inhibition of BPs towards four SULT isoforms, which may provide a new perspective on the toxicity mechanism of BPs and further explain the interference of BPs on endocrine hormone metabolism.

## Introduction

Bromophenols (BPs), a group of brominated compounds, could come from both synthetic and natural sources. With the development of science and technology, BPs are widely used in herbicides, pesticides, wood protectants, and flame retardants, thus leading to frequent contact with such chemical products in daily life (1), and detected in multiple foods such as eggs, fish, milk, and fat. Humans are the last link in the entire chain of exposure suggesting a wide range of exposure pathways and higher concentrations due to the cumulative effect (2).

Many studies have shown that BPs can cause endocrine disruption and other chronic toxicity. For example, 3-BP, as a potential PBT (persistent, bioaccumulative, and toxic) substance, has significant thyroid hormone activity and estrogen effect. 2,4,6-TBP can disrupt the endocrine system (3) *via* interfering with the function of the Ca^2+^ channel in neuroendocrine cells, or it can inhibit the activity of thyroid hormone SULT in human hepatocytes (4). So it is necessary to study the metabolic interference of BPs on endocrine hormones.

Sulfonation as an important phase II metabolism has been known, involved in the metabolism of biomass, drugs and endogenous compounds. Compared with other Phase II enzymes, sulfotransferase (SULT) is regarded as a more vital role in protecting against xenobiotics and in regulating hormone functions during the development of fetal, neonatal, and infant (5–7). In most cases, sulfate conjugation may result in the inactivation of the substrate compounds or increase their water-solubility, thereby facilitating their removal from the body (8). SULTs can catalyze the transfer of the sulfuryl group from 3-phosphoadenosine-5-phosphosulfate (PAPS) to the many substrates such as estradiol and dehydroepiandrosterone (DHEA). 12 kinds of SULT have been found in the human body. Among them, SULT1A1, SULT1A3, SULT1B1, SULT1E1 are more abundant in the human body and metabolize many important endogenous substances (9). SULT1A1 accounts for half of the SULT protein in the liver and is responsible for the metabolism of plane phenols, thyroxine, and estrogen (10). SULT1B1 is the main sulfotransferase expressed in the small intestine, which together with SULT1A3/4 constitutes 67% of the sulfotransferase protein in this tissue (11). Due to a similar structure, its metabolic profile overlaps with that of SULT1A1. SULT1A3 exists in primates with dopamine as the prime substrate. Estrogen sulfotransferase (SULT1E1) is an enzyme that maintains hormone homeostasis and biosynthesis and metabolizes estrogen and iodine adenine.

Several xenobiotics, e.g. polychlorinated biphenyls, halogenated phenol, and dietary polyphenol, have been considered as typical environmental endocrine disruptors, which exist in persistent organic pollutants. However, there is currently no systematic and comprehensive study of BPs on endocrine hormone metabolism. In this study, the inhibitory properties of BPs on SULT were investigated through *in vitro* enzymatic reactions, which can further interpret environmental pollutants to interfere with endocrine hormone metabolism.

## Materials and Methods

### Chemicals and Reagents

Eight BPs (2-BP, 3-BP, 4-BP, 2,4-DBP, 2,5-DBP, 2,6-DBP, 3,5-DBP and 2,4,6-DBP) were purchased from J&K Chemical Ltd. (Beijing, China), with purity over 98%. Recombinant human SULT isoforms were obtained from BD Gentest Corp. (Woburn, MA, USA). P-nitrophenol (PNP) and its sulfate PNP-S, Tris-HCl, and MgCl_2_ were from Sigma-Aldrich (St. Louis, MO, USA), and so is PAPS with a purity of 60%. Acetonitrile was purchased from Tianjin Saifurui Technology Ltd. ultra-pure water was prepared by Millipore Elix 5 UV and Milli-Q Gradient Ultra-Pure Water System. The other reagents were high-performance liquid chromatography (HPLC) grade or the highest grade commercially available.

### Enzyme Activity Assays and Kinetic Study

The incubation mixture (12) with a total volume of 200 μL, contained 100 mM of Tris-HCl buffer (pH=7.4), 5 mM of MgCl_2_, 40 μM of PAPS, SULTs, and PNP. The concentrations of SULT1A1, SULT1A3, SULT1B1 and SULT1E1 were 10 μg/mL, 10 μg/mL, 10 μg/mL and 20 μg/mL, respectively. After 3-min pre-incubation, PAPS was added to initiate the metabolic reaction. The reaction temperature was set to be 37°C, and the reaction time was 30-60 min. 100 μL of ice-cold acetonitrile was added to terminate the reaction. The incubation mixture without BPs was used as a control. After centrifugation at 12,000 rpm, 10 μL of supernatants were analyzed using ultra-performance liquid chromatography (UPLC)-UV instrument. UPLC separation was achieved using a C18 column (4.6*200 mm, 5 μm, Kromasil) at a flow rate of 0.4 mL/min, and the column temperature was 25°C. The mobile phases contained phase A (H_2_O containing 0.5% (v/v) formic acid) and phase B (acetonitrile). Gradient condition was used as followed: initiated at 5% B, increased to 95% over 2 minute after 6 minute, held constant for 6 minutes. The UV detection wavelength was 280 nm. In order to determine the Michaelis-constant (K_m_) of PNP metabolites, incubation mixture (total volume=200 μL) contained BPs (100 μM), Tris-HCl buffer (50 mM, pH=7.4), MgCl_2_ (5 mM), PAPS (4 mM), SULTs and PNP. The corresponding PNP concentrations for SULT1A1, SULT1A3, SULT1B1 and SULT1E1 were 1-400 μM, 0.1-7 mM, 0.1-1 mM and 0.1-1.2 mM, respectively. Incubation and analysis conditions were conducted as described above. The kinetic parameters V_max_ and K_m_ were determined by fitting data to the Michaelis–Menten equation or substrate inhibition equation.

### Preliminary Screening of Inhibition Capability of BPs on SULTs

The incubation mixture (total volume=200 μL) contained 100 μM of BPs, 100 mM of Tris-HCl buffer (pH=7.4), 5 mM of MgCl_2,_ 40 μM of PAPS, SULTs, and PNP. The concentrations of SULT1A1, SULT1A3, SULT1B1 and SULT1E1 are 10 μg/mL, 10 μg/mL, 10 μg/mL and 20 μg/mL. The concentrations of PNP were 40, 1200, 25, 120 μM for SULT1A1, SULT1A3, SULT1B1, SULT1E1 (based on the K_m_ values). Incubation mixture without BPs was used as negative controls. All incubations were carried out in duplicate.

### Half Inhibition Concentration (IC_50_) and Inhibition Kinetics Determination

Half inhibition concentration (IC_50_) was conducted by adding 0 μM to 100 μM BPs. The inhibition kinetics were determined with BPs and PNP covering the K_m_ (for PNP) and IC_50_ values (for BPs). Lineweaver-Burk (L-B) plot was drawn using 1/reaction velocity (v) versus 1/the concentration of PNP ([PNP]) to determine the inhibition kinetic type. In the second plot, inhibition kinetics (K_i_) were calculated by the slopes of the lines in the Lineweaver-Burk plots.

### *In Vitro-In Vivo* Extrapolation (IVIVE)

*In vitro-in vivo* extrapolation (IVIVE) was employed to predict *in vivo* inhibition possibility for BPs towards SULTs by using the following equation:


$${\text{AUC}}_{\text{i}}\text{/AUC}=1+[\text{I}]{\text{/K}}_{\text{i}}$$

AUC_i_/AUC was the predicted ratio of *in vivo* exposure of xenobiotics or endogenous substances with or without the co-exposure of BPs. [I] was the *in vivo* exposure concentration of BPs, and the K_i_ value was *in vitro* inhibition constant. The AUCi/AUC cutoffs of < 1.25 were considered as an inhibition for a significant *in vivo* (13).

### *In Silico* Docking

*In silico* docking was employed to understand the molecular interaction between BPs and SULTs. We constructed the structure of SULT isoforms by homology modeling method with the MODELLER9v14 program. Autodock software (version 4.2) was utilized to dock BPs into the activity cavity of SULT isoforms, respectively. The non-polar hydrogen atoms of SULTs were merged. The grid box was generated with 60 ×60×60 in X, Y, and Z coordinate to cover the entire ligand-binding site. Lamarckian Genetic Algorithm (LGA) method was utilized to study the molecular docking of active domain between BPs and SULTs. The LGA runs were set to 50 runs for each BPs. The best conformation with the lowest docked energy was analyzed for the interactions between BPs and SULTs including hydrogen bonds and hydrophobic contacts.

### Data Analysis

The experimental data were presented as the mean value plus standard deviation (SD). Statistical analysis was carried out using GraphPad Prism 5.0. Comparisons between two groups were performed using a two-tailed unpaired Student’s t-test. Multiple groups were compared using a one-way ANOVA.

## Results

### Kinetic Study and Detection Result

PNP-sulfate was detected at 4.4 min by UPLC, and the substrate PNP eluted at 5.0 min. The metabolism of SULT1A1 to PNP conformed to the substrate inhibition model, and the metabolism of SULT1A3, SULT1B1, SULT1E1 to PNP conformed to the Michaelis–Menten equation (**Supplementary Figure 1**). The kinetic parameters (including K_m_, K_i_) of PNP catalyzed by SULTs were summarized (**Supplementary Table 1**).

### Preliminary Screening of Inhibition Capability of BPs Towards SULTs

**Figure 1:**
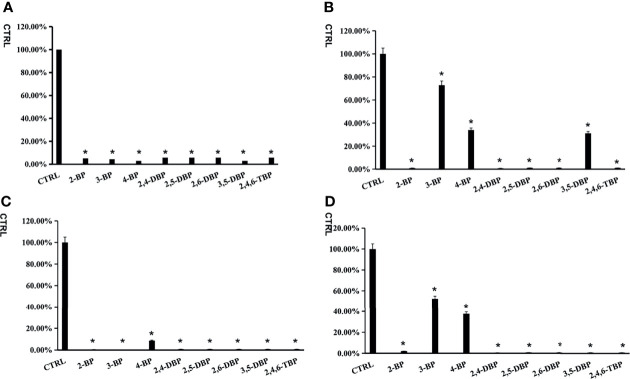
The inhibition screening of bromophenols towards SULT1A1 **(A)**, SULT1A3 **(B)**, SULT1B1 **(C)** and SULT1E1 **(D)**. The data were given as mean value plus S.D. (n=3).*p < 0.05.

All BPs showed an inhibitory effect on the activity of four SULTs (**Figure 1**). From the preliminary screening results, we could find some structural properties of BPs for inhibiting SULTs. The introduction of 2-position bromide substituents increased the inhibitory potential of 3-BP and 4-BP towards SULT1A3 and SULT1E1. Similarly, the introduction of 5-position bromide substituents increased the inhibitory potential of 3-BP towards SULT1A3 and SULT1E1. Furthermore, BPs with 2-substituted bromine atoms exhibited severe inhibition potential towards all four tested SULTs isoforms with an inhibition rate above 80%. 3-BP and 4-BP only showed strong inhibition ability towards SULT1A1 and SULT1B1, but not SULT1A3 and SULT1E1.

### Inhibition Kinetics Determination

**Figure 2:**
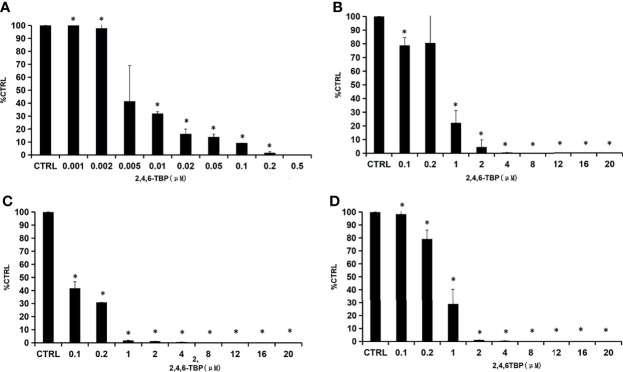
Concentration-dependent inhibition of 2,4,6-TBP towards SULTs. **(A–D)** presents concentration-dependent inhibition of 2,4,6-TBP towards SULT1A1 **(A)**, SULT1A3 **(B)**, SULT1B1 **(C)** and SULT1E1 **(D)**.The data were given as mean value plus S.D. (n=3). *p < 0.05.

**Figure 3:**
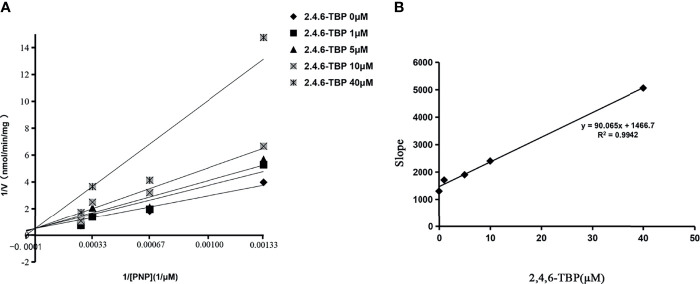
The Lineweaver-Burk plot of 2,4,6-TBP to SULT1A3. In **(A)**, the horizontal axis represents the value of 1/[PNP]. The vertical axis represents 1/V. V is the velocity of the reaction. K_i_ values were calculated using the slopes of **(B)**.

2,4,6-TBP was selected as the representative BPs to determine the inhibition kinetics. As shown in **Figure 2**, the concentration-dependent inhibition of 2,4,6-TBP towards SULT1A1, SULT1A3, SULT1B1, and SULT1E1 was proved. The concentration-dependent inhibition of 2-BP, 2,4-DBP, 2,5-DBP, and 2,6-DBP towards SULT1A1, SULT1A3, SULT1B1, and SULT1E1 was given in **Supplementary Figures 2** **–** **5**. The calculated IC_50_ values for the inhibition of these BPs towards SULT1A1, SULT1A3, SULT1B1, and SULT1E1 were given in **Supplementary Table 2**. Furthermore, the inhibition type and kinetic parameters (K_i_) were determined for SULT1A3 by 2,4,6-TBP. As shown in **Figure 3A**, the intersection point was located in the vertical axis, indicating the competitive inhibition of 2,4,6-TBP towards SULT1A3. The fitting equation of the second plot was y=90.065x+1466.7 for the inhibition of 2,4,6-TBP towards SULT1A3 (**Figure 3B**). Based on this equation, the inhibition kinetic parameter (K_i_) was calculated to be 16.28 µM.

### *In Silico* Docking to Elucidate the Inhibition Mechanism

**Figure 4:**
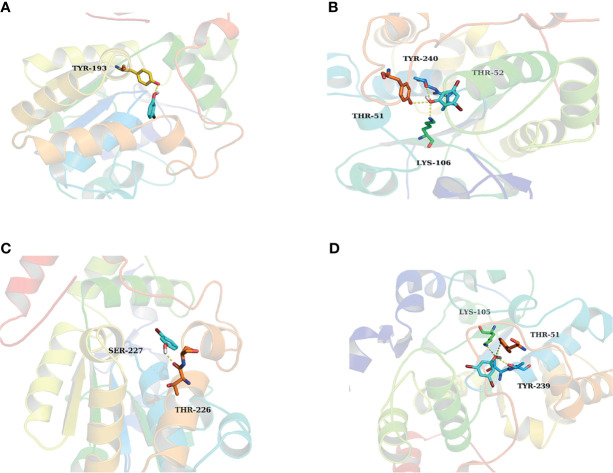
The activity cavity of between 3-BP, 3,5-DBP and SULT1A3, SULT1E1. 3-BP formed 1 hydrogen bond with SULT1A3 **(A)** and SULT1E1 **(C)**, respectively. 3,5-DBP formed 3 hydrogen bonds with SULT1A3 **(B)** and SULT1E1 **(D)**, respectively.

Results of *in silico* docking showed the influence of the introduction of substituted bromine on the inhibition capability. The binding free energy of 3-BP and 3,5-DBP with SULT1A3 were -5.77 kcal/mol and -6.41kcal/mol, respectively, which is consistent with the experimental results that 3,5-DBP exerted stronger inhibition than 3-BP towards SULT1A3. 3-BP formed one hydrogen bond with SULT1A3 (**Figure 4A**), and 3,5-DBP formed three hydrogen bonds with SULT1A3 (**Figure 4B**). Similar results were found in 3-BP and 3,5-DBP towards SULT1E1 (**Figures 4C, D**). Based on these results, we get the conclusion that more hydrogen bonds formation contributed to the stronger inhibition effect of 3,5-DBP than 3-BP towards SULT1A3 and SULT1E1.

## Discussion

In this study, we demonstrated the inhibition characteristics of BPs towards four SULTs isoforms. All of the BPs could inhibit the metabolic activity of four SULTs isoforms. Among them, SULT1A1 and SULT1B1 were the most vulnerable isoforms. 2-substituted bromophenol exhibited severe inhibition potential towards four SULTs. 2,4,6-TBP was selected as a representative BP to determine the inhibition kinetics, including the inhibition type and kinetic parameters (K_i_). Based on the IVIVE equation, the threshold value of 2,4,6-TBP was 1.628 µM. 2,4,6-TBP was regarded as the most abundant BP in the human body. The concentrations of 2,4,6-TBP ranged from 27 ng/g lipid to 81 ng/g (0.08 - 0.24 µM) lipid in serum (14) and detected and present at higher concentrations with a mean concentration of 15.4 ng/g lipid (range: 1.31-316 ng/g lipid) (0.004 - 0.95 µM) in placental tissues (15). Therefore, 2,4,6-TBP seemed to exert a low possibility to inhibit the activity of SULT1A3. However, considering the accumulation of 2,4,6-TBP and its association with other toxic substances, the possibility of 2,4,6-TBP inhibiting SULT1A3 *in vivo* might increase.

It should be noted that there are some studies to demonstrate the substrate properties of BPs for SULTs. For example, a previous study has shown that 2,4,6-TBP is a typical substrate of SULTs (16). UPLC-MS analysis of urine samples has identified the sulfate and glucuronide metabolites of 2-BP (17). Therefore, glucuronidation and sulfation are the main elimination pathways of BPs in humans.

UDP-glucuronosyltransferases (UGTs) and SULTs, the most important phase II metabolic enzymes, are the main endogenous substances that undergo the metabolism catalyzed. For example, both UGTs and SULTs are involved in the metabolic elimination of thyroid hormones (18, 19). The metabolism of estrogen needs the catalysis of SULTs and UGTs (20, 21). Oral administration to 2,4,6-TBP in adult zebrafish and found that reproductive toxicity in addition to perturbed gonadal morphology when exposed to high levels of food at 3300 μg/g dw (22). Although the *in vivo* concentrations measured in the present study are lower than the *in vivo* threshold in this study, when both of these pathways are inhibited, the metabolism of estrogen may be significantly disturbed. Therefore, due to the inhibitory effect of BPs on UGTs and SULTs, attention should be paid to the metabolic homeostasis of endocrine hormones.

## Conclusion

This study demonstrated the inhibition of BPs towards SULT1. Our results showed that SULT1A1 and SULT1B1 were demonstrated to be the most vulnerable SULT isoforms towards BPs’ inhibition. Structure-inhibition activity relationship analysis showed that 2-substituted bromophenol exhibited severe broad inhibition potential towards four SULTs, and the addition of 5-position bromide in BPs can increase the inhibition capability. *In silico* docking, we get the conclusion that more hydrogen bonds formation contributed to the stronger inhibition effect of 3,5-DBP than 3-BP towards SULT1A3 and SULT1E1. *In vitro-in vivo* extrapolation (IVIVE) was performed to predict the threshold value of 2,4,6-TBP to induce *in vivo* inhibition of SULTs. These results may interpret BPs to interfere with endocrine hormone metabolism.

## Data Availability Statement

The original contributions presented in the study are included in the article/**Supplementary Material**. Further inquiries can be directed to the corresponding author.

## Author Contributions

ZL contributed to the study’s conception and design. Material preparation, data collection, and analysis were performed by ZD and FZ. The first draft of the manuscript was written by ZD and YL. JX commented on previous versions of the manuscript. All authors read and approved the final manuscript.

## Funding

This work was supported by the Educational Department of Liaoning Province (No. JYTZD2020003).

## Conflict of Interest

The authors declare that the research was conducted in the absence of any commercial or financial relationships that could be construed as a potential conflict of interest.

## Publisher’s Note

All claims expressed in this article are solely those of the authors and do not necessarily represent those of their affiliated organizations, or those of the publisher, the editors and the reviewers. Any product that may be evaluated in this article, or claim that may be made by its manufacturer, is not guaranteed or endorsed by the publisher.
