# Supplementary material for: The Environmental Pollutant Bromophenols Interfere With Sulfotransferase That Mediates Endocrine Hormones
Source: Front Endocrinol (Lausanne). 2022 Jan 7;12:814373. doi: 10.3389/fendo.2021.814373 (PMC8777265; doi:10.3389/fendo.2021.814373)

Supplementary Material

Supplementary Table 1 The metabolic kinetics values (K_m_, V_max_)of PNP catalyzed by SULTs.

|  | Km （μM） | Vmax （nmol/min/mg protein） |
| --- | --- | --- |
| SULT1A1 | 28 | 53324 |
| SULT1A3 | 1207 | 21040 |
| SULT1B1 | 23 | 1839 |
| SULT1E1 | 118 | 2705 |

Supplementary Table 2 IC_50_ (μM) values of BPs towards SULTs.

|  | SULT1A1 | SULT1A3 | SULT1B1 | SULT1E1 |
| --- | --- | --- | --- | --- |
| 2-BP | 1.035 | 8.875 | 1.074 | 25.92 |
| 2,4-DBP | 1.026 | 25.49 | 4.223 | 3.239 |
| 2,5-DBP | 1.243 | 12.88 | 5.425 | 1.764 |
| 2,6-DBP | 1.075 | 3.418 | 1.503 | 1.606 |
| 2,4,6-TBP | 1.015 | 4.019 | 1.059 | 6.060 |


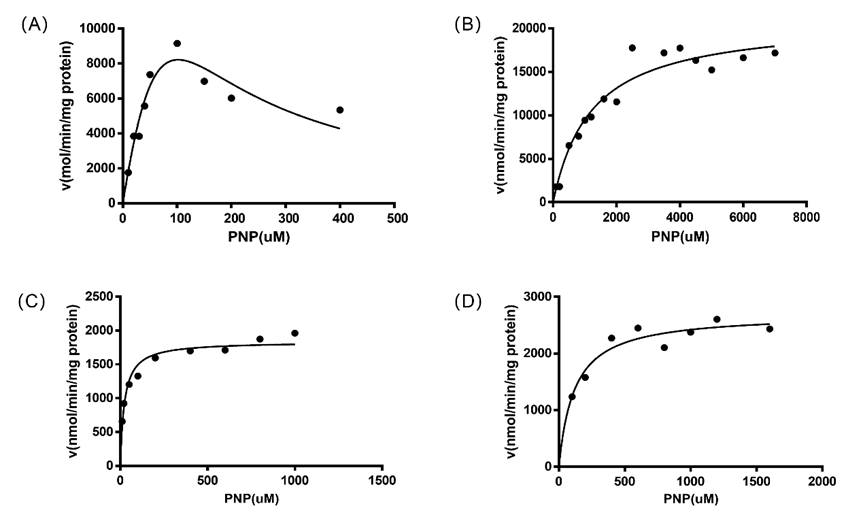
Supplementary **Figure 1** Kinetic parameters (K_m_, V_max_) of PNP catalyzed by SULTs.

Supplementary **Figure 2** Concentration-dependent inhibition of 2-BP towards four SULT isoforms. Parallel samples were made, and the average values were used to draw the graph. **A-D** presents concentration-dependent inhibition of 2-BP towards SULT1A1, SULT1A3, SULT1B1, SULT1E1.


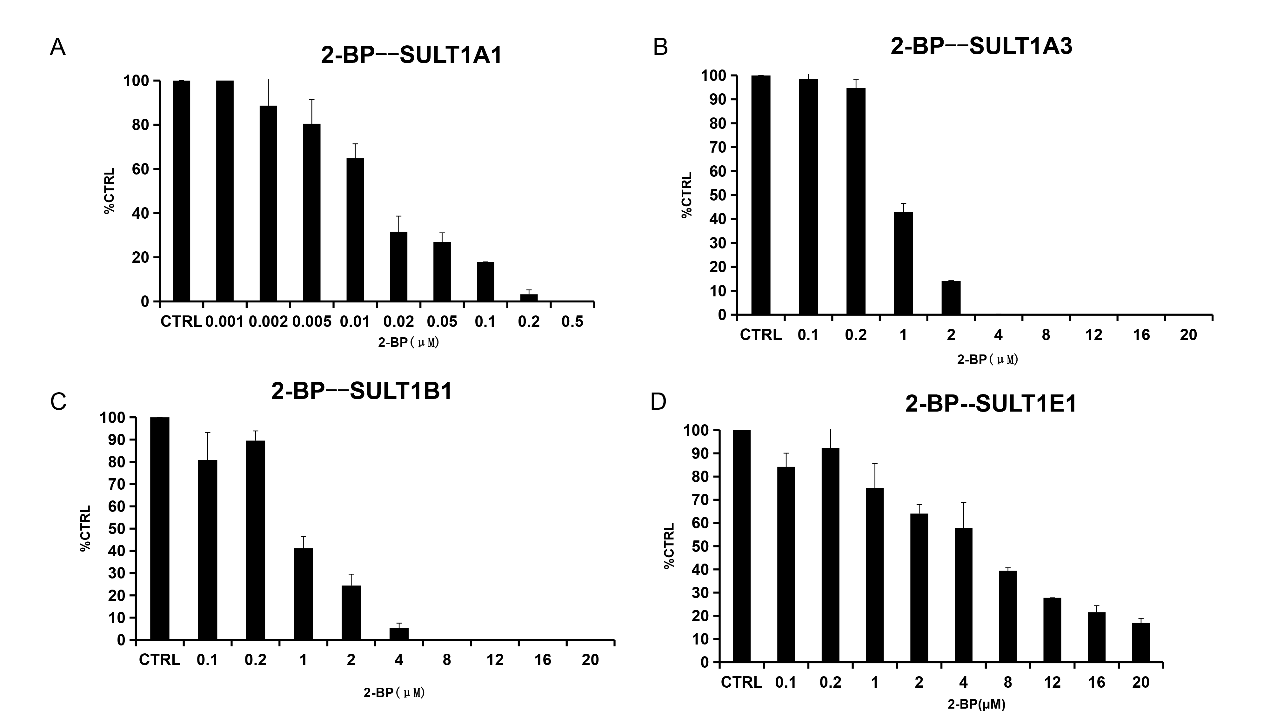


Supplementary **Figure 3** Concentration-dependent inhibition of 2,4-DBP towards four SULT isoforms. Parallel samples were made, and the average values were used to draw the graph. **A-D** presents concentration-dependent inhibition of 2,4-DBP towards SULT1A1, SULT1A3, SULT1B1, SULT1E1.


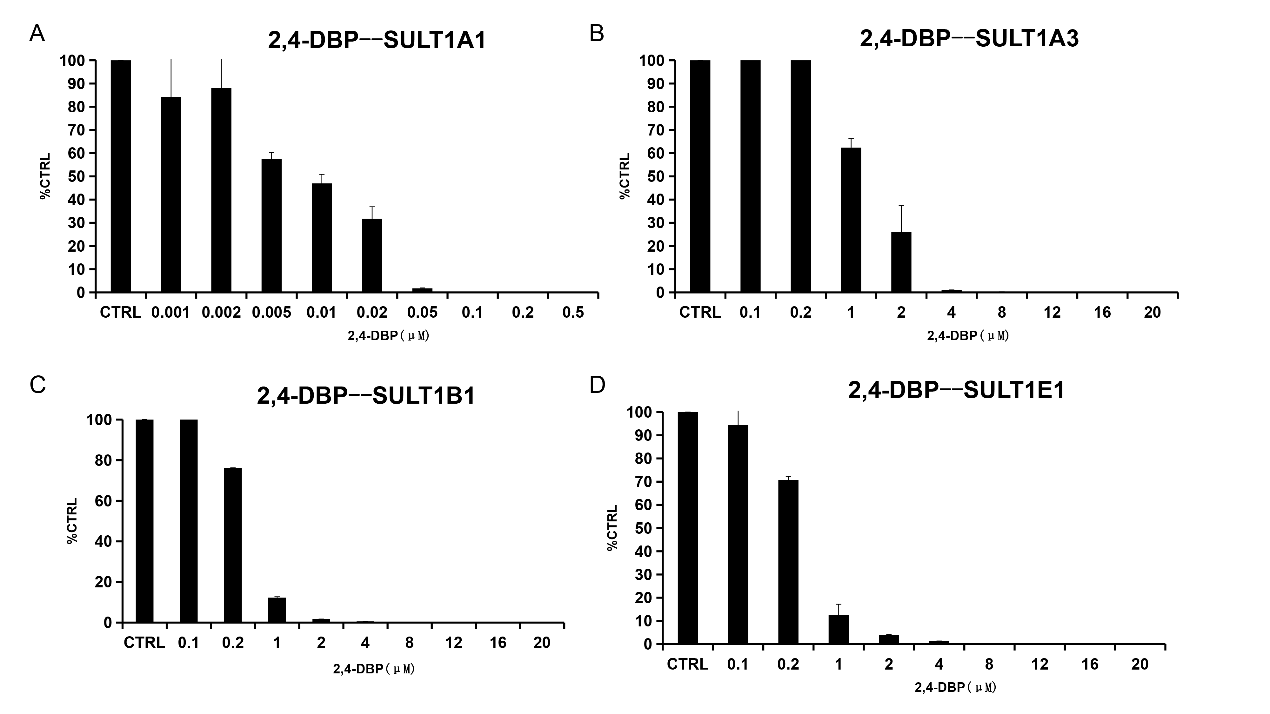


Supplementary **Figure 4** Concentration-dependent inhibition of 2,5-DBP towards four SULT isoforms. Parallel samples were made, and the average values were used to draw the graph. **A-D** presents concentration-dependent inhibition of 2,5-DBP towards SULT1A1, SULT1A3, SULT1B1, SULT1E1.


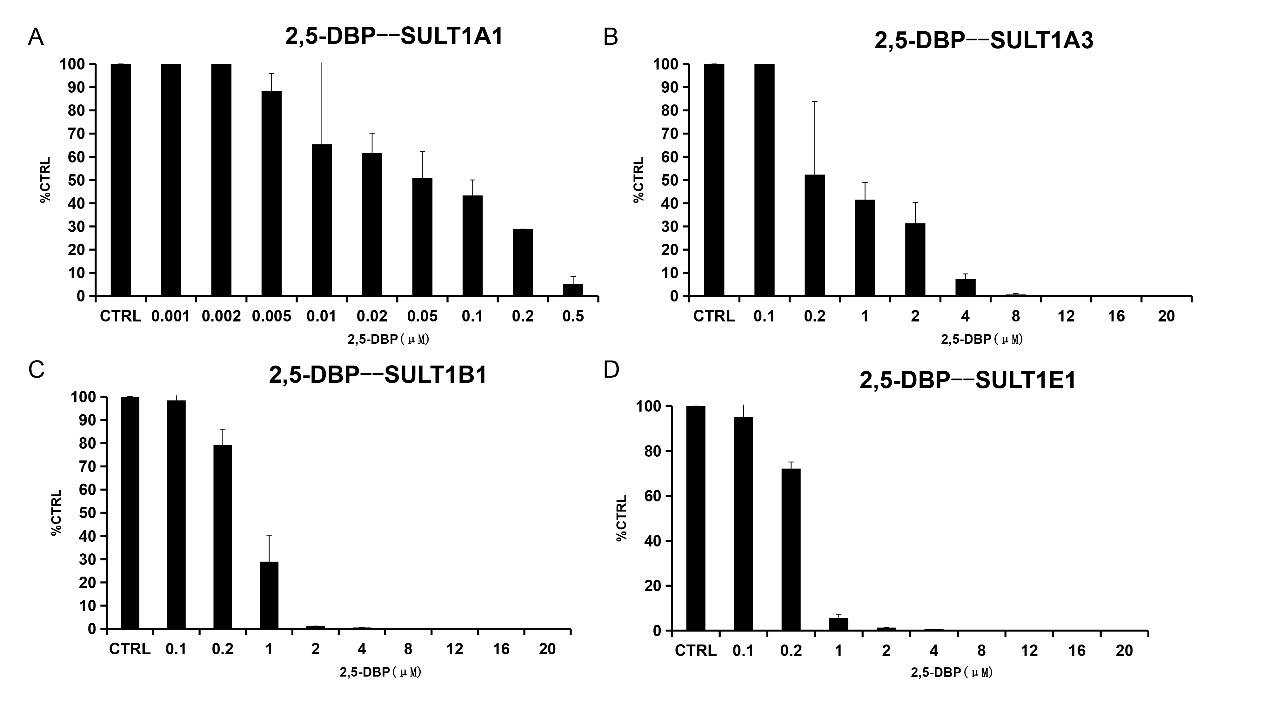


Supplementary **Figure 5** Concentration-dependent inhibition of 2,6-DBP towards four SULT isoforms. Parallel samples were made, and the average values were used to draw the graph. **A-D** presents concentration-dependent inhibition of 2,6-DBP towards SULT1A1, SULT1A3, SULT1B1, SULT1E1.


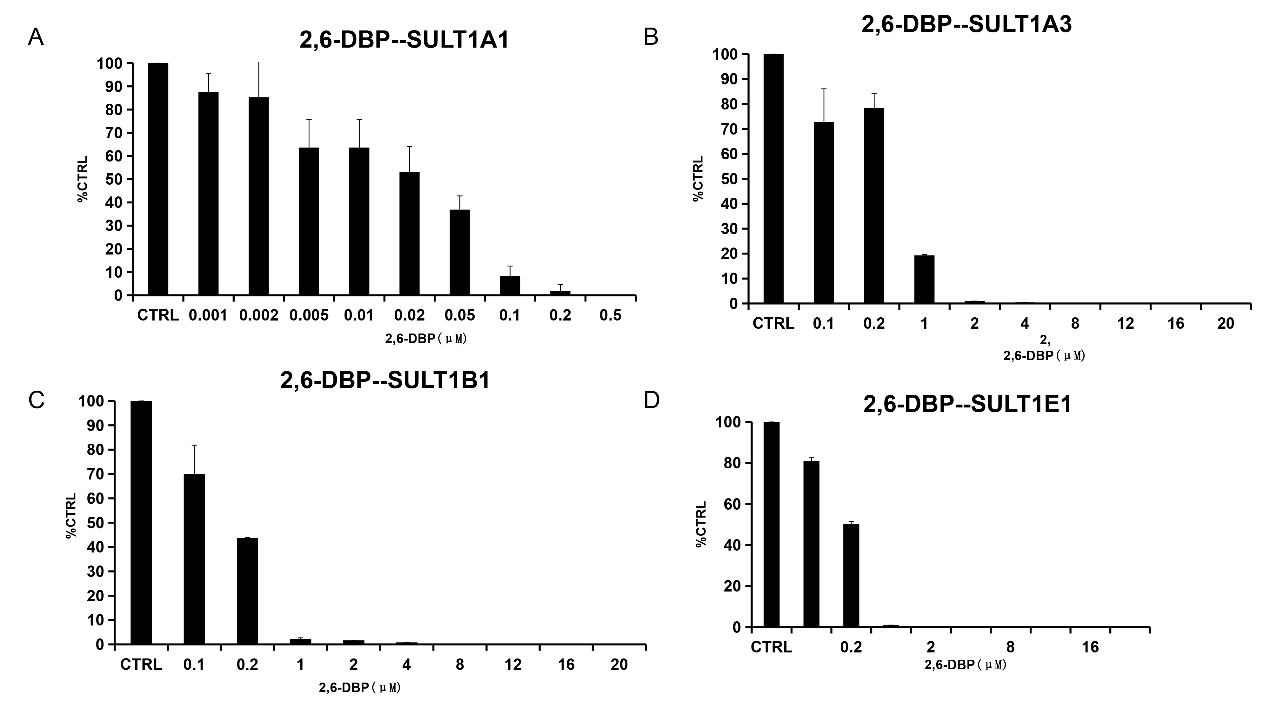

Supplement: Supplementary file 1 [file DataSheet_1.docx]
